# Immune Checkpoint Inhibitor Dosing: Can We Go Lower Without Compromising Clinical Efficacy?

**DOI:** 10.1200/JGO.19.00142

**Published:** 2019-07-26

**Authors:** Alex Renner, Mauricio Burotto, Carlos Rojas

**Affiliations:** ^1^ University of Chile Clinical Hospital, Santiago, Chile; ^2^ Los Andes University, Santiago, Chile; ^3^ Bradford Hill Clinical Research Center, Santiago, Chile

## Abstract

In just a few years, immune checkpoint inhibitors have dramatically changed the landscape in oncology, offering durable responses and improved survival for many patients across several tumor types. With more than 3,300 new agents in the immuno-oncology pipeline plus a wide array of combinations being studied, it seems this new era is just getting started. These advances come with a significant caveat: most of the world population does not have access to their benefits, because the yearly cost of a novel anticancer medication can routinely exceed $100,000. There is a large amount of data showing that checkpoint inhibitors have significant activity at doses much lower than those currently approved. We review the evidence for reduced drug dosing as a strategy to increase the number of patients who can be treated and what would be needed to further validate this approach.

## LOOKING FOR THE OPTIMAL DOSE

Maximum tolerated dose (MTD), a dose selection strategy that derives from cytotoxic agent development, has proven challenging for checkpoint inhibitors and molecularly targeted agents because there is no clear dose-response relationship, and the identification of an MTD may not be a realistic objective. In fact, in studies performed with pembrolizumab,^1^ ipilimumab,^2^ atezolizumab,^3^ durvalumab,^4^ and nivolumab,^5^ the investigators did not identify an MTD. In this scenario, we review the available data for two anti–programmed death-1 (PD-1) agents that have extensive published data on dose selection, dose-response, and comparative clinical efficacy for different dosing strategies: pembrolizumab and nivolumab.

**CONTEXT**

**Key Objective**
Is it possible to treat patients with a lower dose of nivolumab or pembrolizumab without affecting treatment efficacy?
**Knowledge Generated**
Both drugs have shown similar response rates in a wide range of doses, some of them much lower than currently approved schedules. This could be explained because the programmed death-1 receptor reaches maximum occupancy at low doses for these agents, translating into a flat exposure-response curve, where increasing the dosage does not lead to an increase in tumor response.
**Relevance**
We need prospective data to validate this strategy, which could lead to much wider access to these potentially life-saving drugs, especially in resource-constrained countries.

## PEMBROLIZUMAB

Pembrolizumab is a humanized immunoglobulin G4 monoclonal antibody, directed against the PD-1 receptor, antagonizing the interaction between it and its ligands, programmed death-ligand 1 (PD-L1) and PD-L2, leading to an increased antitumor immune response. The key metric of response to anti-PD1 antibodies is cytokine production, which in the case of pembrolizumab was determined by measuring interleukin-2 (IL-2) production by T cells. Pharmacodynamic saturation, defined as the inability of additional pembrolizumab to increase IL-2 production, was demonstrated even at the lowest tested dose (0.3 mg/kg) in cynomolgus monkeys.^6^ The same pharmacodynamic metric was used in human studies, the most significant being KEYNOTE-001,^1^ a phase I study, which conducted a 3 + 3 dose escalation enrolling patients at 1, 3, and 10 mg/kg doses. No dose-limiting toxicities were observed during dose escalation, and no MTD was reached. The researchers also performed an ex vivo IL-2 stimulation test to determine PD-1 receptor saturation, showing a 95% target engagement with a single dose of 1 mg/kg; higher doses increased serum concentration with no meaningful change in receptor saturation, and even lower doses still showed elevated median target engagement: approximately 90% for 0.5 mg/kg and 80% for 0.2 mg/kg.^7^ As with any ex vivo study, these results cannot be simply extrapolated to the clinical setting but give us a potential mechanistic explanation for the flat exposure response observed in later stages of drug development.

In addition to single-dose pharmacokinetics, there is a reduction in pembrolizumab clearance over time, which was not identified in the initial models; current data confirm a decrease in clearance between 20% and 30% at the steady state compared with the clearance after the first dose,^8^ which is now recognized in the US product labeling. Most of this reduction takes place during the first 5 months of treatment. Although the exact mechanisms are still being studied, it has been shown to be related to larger baseline tumor size, higher Eastern Cooperative Oncology Group score, and higher tumor response to treatment. As clearance decreases, plasmatic levels increase over time; thus, the receptor saturation studies are probably underestimating receptor saturation in standard clinical practice, with longer treatment durations. A consolidated exposure-response model using integrated data from KEYNOTE-001, -002, and -006 was developed by Chaterjee et al^9^ showing there is no significant difference in tumor response at doses ranging from 1 to 10 mg/kg once every 3 weeks.

Several clinical trials confirmed the fact that increasing the dose has no statistically significant effect on tumor response. Robert et al^10^ compared pembrolizumab at doses of 2 mg/kg once every 3 weeks and 10 mg/kg once every 3 weeks in 173 patients with advanced melanoma; the objective response rate (ORR) was identical (26%) for both groups. Ribas et al^11^ compared the same doses of 2 mg/kg once every 3 weeks and 10 mg/kg once every 3 weeks in 540 patients with ipilimumab-refractory melanoma, showing that progression-free survival by independent central review was similar (2.9 *v* 2.9 months), and ORR by independent central review was also similar (21% *v* 26%).

Unfortunately, no clinical trials have been reported with doses lower than 2 mg/kg once every 3 weeks, which is the dose with which pembrolizumab was initially registered. Table 1 lists the ORR for pembrolizumab across different dosing schedules. A few years after the first regulatory approval, Merck pursued and approved a flat dose of 200 mg once every 3 weeks in first-line treatment of non–small-cell lung cancer (NSCLC), leading to unnecessarily high doses for patients with lower body weight. The economic impact of this change has been calculated^12^ as an additional expenditure of more than $800 million per year, just for the NSCLC indication in the United States. In addition, the company has steadily raised the price per milligram.^13^ The combined net effect of these measures is increasing financial toxicity in conjunction with a likely absent improvement in clinical efficacy.

**TABLE 1:**
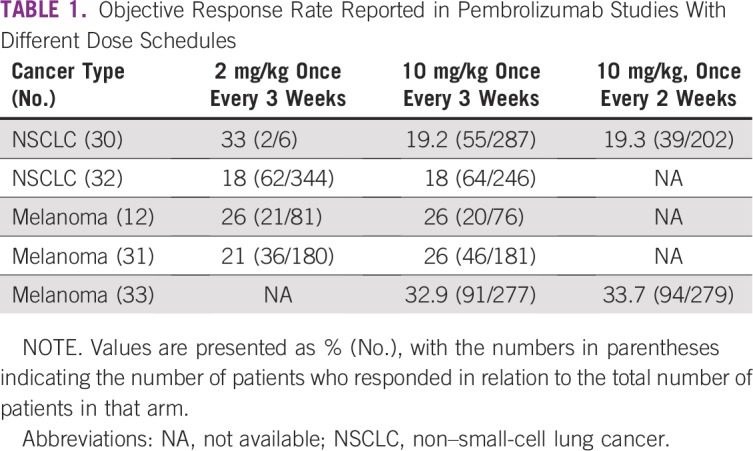
Objective Response Rate Reported in Pembrolizumab Studies With Different Dose Schedules

## NIVOLUMAB

Nivolumab, similar to pembrolizumab, is a humanized immunoglobulin G4 monoclonal antibody directed to the PD-1 receptor, blocking it from binding to its ligands. It has a high affinity to its target, because in vitro studies show that 0.04 μg/mL of nivolumab, which is below enzyme-linked immunosorbent assay–detectable serum levels of 1.2 μg/mL, is enough to occupy more than 70% of PD-1 receptors on T cells.^5^ A dose-ranging phase Ib study confirmed this high affinity, showing that peripheral PD-1 receptor occupancy was already saturated at a 0.3-mg/kg dose.^14^ The Food and Drug Administration (FDA) clinical pharmacology review^6^ mentions the trough concentration for the dose of 3 mg/kg once every 2 weeks is more than 16 µg/mL, which is more than 160 times the half maximal effective concentration for receptor binding.

It is not surprising, then, that no relevant exposure efficacy has been found in clinical trials using doses over 0.1 mg/kg. Topalian et al^15^ reported results for 107 patients with advanced melanoma who were treated with nivolumab at doses ranging from 0.1 to 10 mg/kg, and there seemed to be no exposure-efficacy relationship for ORR, varying from 20.0% (10 mg/kg) to 41.2% (3 mg/kg), whereas the lowest dose of 0.1 mg/kg still showed high activity, with an ORR of 35.3%. Furthermore, dose escalation was used on progression, increasing from 0.1 to 1.0 mg/kg in five patients and from 0.3 to 1.0 mg/kg in six patients, with no responses observed in any of these patients. Table 2 lists the exposure-response ORR observed for nivolumab across several tumors and dosing schedules. As in Table 1, these data must be interpreted with caution, because regimens were usually tested in limited numbers of patients; as an example, the response rate for the 1.0 mg/kg regimen in NSCLC was only 6%, but because this was tested in only 18 patients with one of them showing response, the 95% CI was 0.1 to 27, and we do not have additional data that could explain this result, such as PD-1 expression for individual patients.

**TABLE 2:**
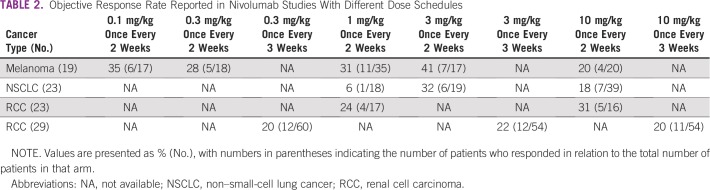
Objective Response Rate Reported in Nivolumab Studies With Different Dose Schedules

Bajaj et al^16^ looked into overall survival exposure response for patients with melanoma receiving regimens of 0.1, 0.3, 1, 3, and 10 mg/kg once every 2 weeks, reporting that time-averaged nivolumab concentration after the first dose was not a predictor of overall survival. Similarly, for lung cancer, Feng et al^17^ reviewed results for 647 patients with NSCLC who received nivolumab doses ranging from 1 to 10 mg/kg once every 2 weeks; they concluded that nivolumab exposure was not associated with overall survival in either squamous or nonsquamous tumors.

Nivolumab was initially approved with a dosage of 3 mg/kg once every 2 weeks. Later, fixed-dose nivolumab regimens of 240 mg once every 2 weeks and 480 mg once every 4 weeks were approved by the FDA for most of the current indications. The approval of 480 mg once every 4 weeks was not based on any new data, but relied solely on pharmacokinetic simulation, dose-response and exposure-response relationships, and clinical safety data. The validity of this in-silico strategy to approve a new dosing regimen without clinical efficacy is debatable; computer modeling has several advantages compared with in vivo studies (significantly lower cost and lower time to produce results, among others) but it is still an area undergoing validation in oncology. For nivolumab, the simulation results showed trough concentrations were 15.6% lower with 480 mg once every 4 weeks compared with 3 mg/kg once every 2 weeks. How is it, then, that no efficacy data were requested, given this finding? The regulatory explanation by the FDA was described in a recent publication by Bi et al,^18^ asserting that “efficacy bridging” is adequate in this scenario, given that a flat dose-efficacy relationship was present in several tumors for a large (100-fold) dose range, from 0.1 up to 10 mg/kg; therefore, it is unlikely that a 15.6% concentration decrease will have any effect on efficacy. In that context, it would be fair to ask just how far this efficacy-bridging argument can be taken for lower concentrations and whether in-silico studies for a lower-dose schedule may be considered valid by regulatory authorities.

## CLINICAL EXPERIENCE WITH LOWER-DOSE IMMUNOTHERAPY

Outside the industry-sponsored phase I studies we have discussed, there is little clinical data with lower doses of these agents. We do have a recent retrospective report from Yoo et al^19^ showing results for 47 patients with stage IIIB to IV NSCLC, 15 of whom received a flat dose of nivolumab 100 mg once every 3 weeks and three of whom received a flat dose of 20 mg once every 3 weeks, whereas 29 patients received the standard 3 mg/kg dose once every 2 weeks. PD-L1 by immunohistochemistry was positive (> 1%) in 31% of patients in the standard-dose group and 22% in the low-dose group. ORR in the low-dose group was 16.7% and 13.8% in the high-dose group. Stable disease was 22% in the low-dose group, whereas it was 10.3% in the standard-dose group. The small number of patients, differing baseline characteristics, and retrospective nature of this report do not allow a valid efficacy comparison among different dosing strategies, but it does show clinical activity with lower doses of nivolumab, which is consistent with the data we have previously reviewed.

## CONCLUSION

Financial toxicity of newer oncology drugs has become a considerable issue for patients and health systems, with access being limited in many countries because of cost. As real-world examples from South America, less than 5% of the population has coverage for PD-1 checkpoint inhibitors in Peru and less than 10% in Chile, the richest country in the region. This problem is not restricted to South America, of course, because even the United States, the country with the world’s largest per capita drug spending, is facing the issue of spiraling drug costs.^20^ Being potentially life-saving drugs, it is urgent that this barrier be reduced and more patients can benefit from them. We believe there is a substantial body of data to back the hypothesis that both pembrolizumab and nivolumab have significant efficacy at much lower doses than those approved by regulatory agencies, because their therapeutic window is much broader than traditional chemotherapy. This would have to be tested in a prospective, randomized fashion. It is unlikely the pharmaceutical industry will be interested in such a subject; therefore, either independent governmental institutions, universities, or collaborative groups would have to take on this challenge, with the potential help of oncology nongovernmental organizations. The impact for our patients could be huge, as we can see in Table 3.

**TABLE 3:**
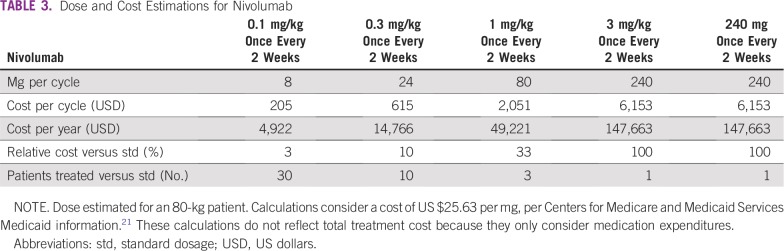
Dose and Cost Estimations for Nivolumab
